# A search for tiny dragons (*Dracunculus medinensis* third-stage larvae) in aquatic animals in Chad, Africa

**DOI:** 10.1038/s41598-018-37567-7

**Published:** 2019-01-23

**Authors:** Christopher A. Cleveland, Mark L. Eberhard, Alec T. Thompson, Kayla B. Garrett, Liandrie Swanepoel, Hubert Zirimwabagabo, Tchonfienet Moundai, Philippe T. Ouakou, Ernesto Ruiz-Tiben, Michael J. Yabsley

**Affiliations:** 1 Southeastern Cooperative Wildlife Disease Study, Veterinary Medicine, University of Georgia, 589 D.W. Brooks Dr., Athens, GA 30601 United States; 2 Warnell School of Forestry and Natural Resources, University of Georgia, 180 E. Green St., Athens, GA 30602 United States; 3 Independent, Retired, Snellville, GA 30339 United States; 4 The Carter Center, 453 Freedom Pkwy NE, Atlanta, GA 30307 United States; 5 Ministry of Public Health, N’Djamena, Chad

## Abstract

*Dracunculus medinensis*, or human Guinea worm (GW), causes a painful and debilitating infection. The global Guinea Worm Eradication Program (GWEP) has successfully reduced human GW cases from 3.5 million in 21 countries in 1986 to only 30 cases in three remaining countries in 2017. Since 2012, an increase in GW infections in domestic dogs, cats and baboons has been reported. Because these infections have not followed classical GW epidemiological patterns resulting from water-borne transmission, it has been hypothesized that transmission occurs via a paratenic host. Thus, we investigated the potential of aquatic animals to serve as paratenic hosts for *D*. *medinensis* in Chad, Africa. During three rainy and two dry season trips we detected no GW larvae in 234 fish, two reptiles and two turtles; however, seven GW larvae were recovered from 4 (1.4%) of 276 adult frogs. These data suggest GW infections may occur from ingestion of frogs but the importance of this route is unknown. Additional studies are needed, especially for other possible routes (e.g., ingestion of fish intestines that were recently shown to be a risk). Significantly, 150 years after the life cycle of *D*. *medinensis* was described, our data highlights important gaps in the knowledge of GW ecology.

## Introduction

Guinea Worm Disease (GWD), caused by the parasitic nematode *Dracunculus medinensis*, is a painful and debilitating disease of people^1,2^. The global campaign to eradicate GWD has been an international success and national Guinea Worm Eradication programs (GWEPs) have reduced the number of human cases from 3.5 million annually in 21 countries to only 30 cases in three remaining countries in 2017^2^. In Chad, infections in dogs, and more recently, cats, have been recorded^2^. Notably, these infections do not typically follow the classic epidemiology of GWD in humans wherein infection occurs from drinking water containing infected copepods. The result of such transmission typically results in a clustering of cases nearby a single water source, which has not been observed in dog or cat cases. While it is still possible that animals may become infected via ingestion of infected copepods in water, an alternate route was hypothesized; fish or frogs may be playing a role in transmission of *D*. *medinensis*^3^. This hypothesis is further supported by the dearth of human cases in many areas where a high incidence of dog infections occur^3^. Furthermore, previous experimental work confirmed that frogs can serve as paratenic hosts for *D*. *medinensis*, that fish may act as short-term transport hosts, and that both routes (frog and fish) can result in infection of a definitive host^4,5^. In 2016, for the first time, a natural infection of an amphibian host from the Sarh region of Chad was reported providing field evidence that amphibians may be involved in transmission of *D*. *medinensis*^6^.

The purpose of this study was to better understand the possible role of aquatic animals (amphibians, fish, and reptiles) as paratenic hosts for *D*. *medinensis* in Chad, Africa. These data are critical for the development of new interventions aimed at interrupting Guinea worm transmission, especially now since the numbers of infections in dogs and cats have increased annually since 2012^2^. Our primary objective was to determine the prevalence of *D*. *medinensis* third-stage (L3) infectious larvae in aquatic animals, with particular attention paid to those species that are likely consumed by humans, those fed to dogs or cats by people, or those that reside in ponds in close proximity to villages where a dog infection or human case has been reported. We also attempted to collect information on which frog species were consumed by humans, the season in which frogs were harvested, and whether or not frogs or fish were fed to dogs and cats.

## Materials and Methods

### Study location and animal acquisition

**Figure 1:**
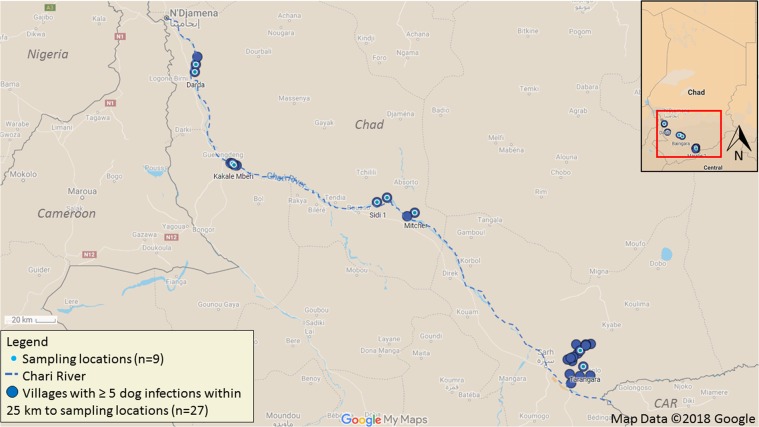
Map of nine sampling site locations and villages within 25 km reporting five or more dog infections in Chad, Africa during 2016–2018 for surveys of *D*. *medinensis* third-stage larvae (L3) in amphibians and fish.

Surveys for *D*. *medinensis* L3s in aquatic hosts from Chad occurred during five trips during 2016–2018. Regions of Chad reporting human cases and dog infections were prioritized for sampling and included Sarh, Bousso, Guelengdeng, and N′Djamena (Fig. 1). Amphibians were captured by local villagers either by hand or through the use of submersible nets baited with fish tissue. Fish were typically purchased from local fishermen either at the pond or river locale being sampled, or from nearby outdoor markets. Reptiles included in the sampling were provided by local villagers.

### Necropsy and sample collection

**Figure 2:**
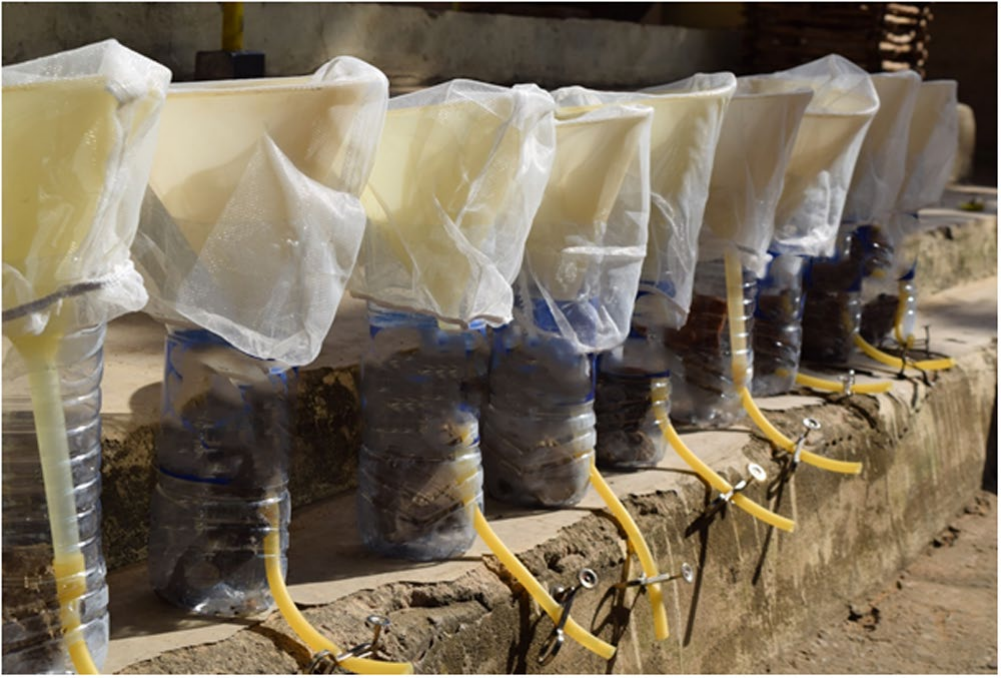
Modified Baermann funnels for sedimentation and recovery of suspect *D*. *medinensis* larvae from muscle tissues of fish and amphibians in Chad, Africa (photo courtesy of C.A. Cleveland).

Necropsies were performed and gastrointestinal (GI) tracts were removed because L3s should only be present in non-GI tissues and muscles^4,5^. Small tissue samples (<1 cm) were taken from every animal and preserved in 70% ETOH for molecular species identification (as described below). For animals <10 cm in length, blunt dissections in petri dishes were performed. Skin was removed and muscle tissue was teased apart using forceps and needle tools. The tissue was allowed to soak in water for at least four hours to allow migration of larvae out of tissue. After four hours, water in petri dishes was observed under a dissecting microscope (30x magnification; StereoZoom 4; Optek, USA) for characteristic sinusoidal movement of larvae. For animals >10 cm in length, after GI and skin removal, samples were minced using a metallic mincer that would render muscle into <0.5 cm diameter pieces (LEM Products, West Chester, Ohio). This tissue was then placed into a mesh screen inside a Baermann funnel with enough water so that all tissue was submerged, and sedimentation of potential nematode larvae was allowed to occur for at least four hours (Fig. 2). At four hours, 5 ml of fluid from the funnel was collected into a petri dish and examined as described above. If the first 5 ml were negative for suspect nematode larvae, two subsequent draws of 5 ml were examined. Any larvae that were morphologically similar to *Dracunculus* were preserved in 70% ETOH for subsequent molecular analyses. Morphological characteristics which constituted subsequent molecular analyses were as follows: an overall length of approximately 0.5–0.7 mm, sinusoidal movement, striated cuticle, and a blunt, trifid tail^6^.

### Molecular identification of host and suspect *Dracunculus* larvae

All suspect samples were observed under a compound microscope for defining characteristics of *D*. *medinensis* L3s, such as a trilobed tail, striated cuticle, and approximate length of 0.581–0.643 mm^7^. Morphologically-compatible larvae and tissue samples were placed in a 0.5 ml microcentrifuge tube and any residual ethanol allowed to evaporate for 12 hours. DNA was extracted using a commercial DNA extraction kit (DNeasy, QIAGEN, Valencia, California) following manufacturer’s instructions for tissue. However, larvae were allowed to incubate at 56 °C with proteinase K and Buffer ATL for six-eight hours to ensure full digestion. For species identification of animal samples, the 16 s rRNA gene target was amplified for fish^8^ and a portion of the cytochrome-*c* oxidase I (COI) gene was amplified for amphibians^4^. To identify larvae, a partial (COI) gene target was amplified using a cocktail of six M13-tagged primers^9^. Amplicons were purified from a 0.8% agarose gel stained with gel red (Biotium Inc., Hayward, California, USA) using a commercial gel-purification kit (QIAGEN). Bi-directional Sanger sequencing was conducted on amplicons at the University of Georgia Genomics Facility (Athens, Georgia) or Genwiz (South Plainfield, New Jersey). Chromatograms were analyzed in Geneious R7 (Auckland, New Zealand) and consensus sequences were compared to sequences in the GenBank database.

### Identification of frog use by villagers

**Figure 3:**
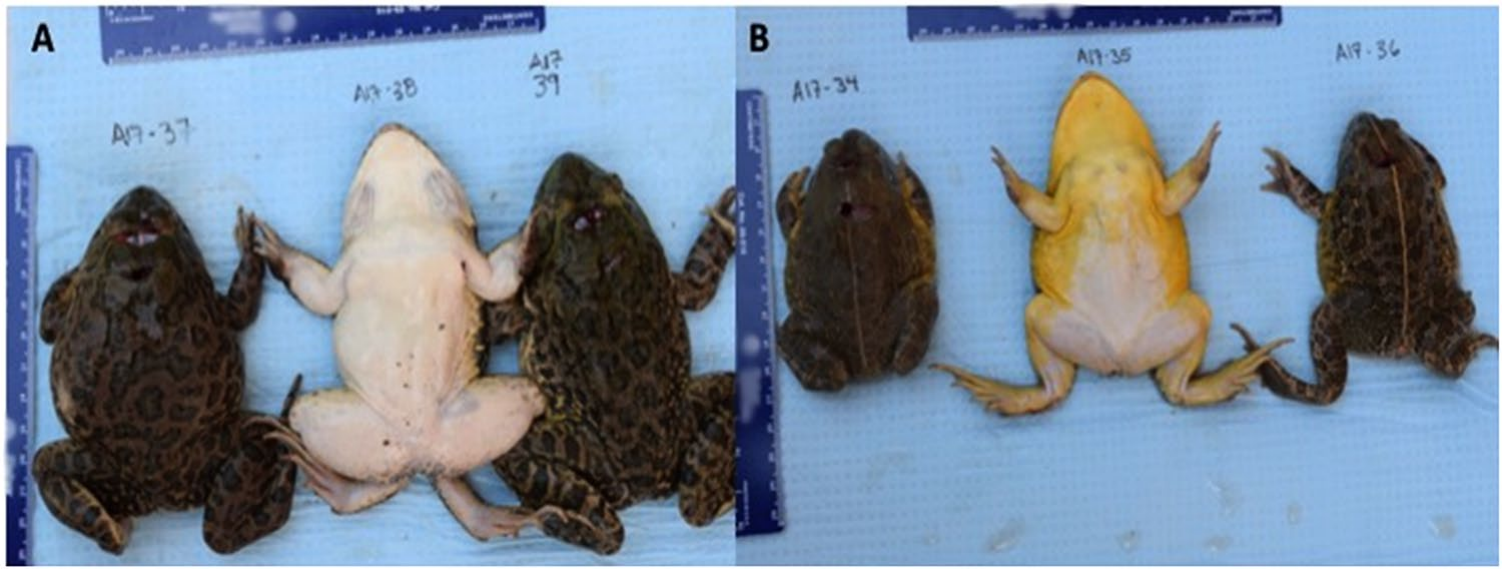
Specimens of *Hoplobatrachus occipitalis* (**A**) and *Pyxicephalus edulis* (**B**) sampled for the presence of *D*. *medinensis* third-stage larvae (L3) from Chad, Africa and shown to villagers during surveys of frog catching and consumption (photo courtesy of C.A. Cleveland).

Where possible, local villagers were queried regarding use and consumption of amphibians as food sources. These questions were as follows: (1) Do you catch and eat frogs?; (2) If you catch frogs, during what season and how?; (3) Can you describe the frogs you catch?; (4) Are frogs cooked or eaten raw?; (5) Do you own a cat or dog, and if so, do you feed them frogs? If individuals stated that they captured frogs, they were shown photos of *Hoplobatrachus occipitalis* (African Crowned bullfrog locally referred to as black-backed, white-bellied river frog) and *Pyxicephalus edulis* (Giant Bullfrog locally referred to as black-backed, yellow-bellied frog) and asked to identify which frogs they capture and during which time of the year (Fig. 3).

### Ethical approval and informed consent

All animal procedures were reviewed and approved by the University of Georgia’s Institutional Animal Care and Use Committee (A2016 07–024). In addition, all methods were performed in accordance with the relevant guidelines and regulations within the aforementioned University of Georgia’s Institutional Animal Care and Use Committee (A2016 07–024).

## Results

During the five trips, we sampled 234 individual fish representing 18 different species, 276 individual amphibians representing six species, two African helmeted turtles (*Pelomedusa* sp.), and two Nile Monitor lizards (*Varanus niloticus*) (Table 1). No *D*. *medinensis* L3s were detected in the tissues of any fish, turtles, or Nile monitor lizards. Of particular note, when skinning the monitor lizards, we found large subcutaneous nematodes on the ventral surface extending along the intercostal muscles and into the thoracic cavity. Previous work had suggested that *Varanus* spp. could be hosts for adult *D*. *medinensis*^10^; however, the morphology of these nematodes clearly indicated they were not *Dracunculus* and molecular characterization using the same primers as used for larvae showed these nematodes were most similar to *Ochoterenella* spp. (Onchocercidae).

**Table 1:** Number and species of fish, amphibians, and reptiles surveyed for *D*. *medinensis* third-stage infectious larvae in Chad, Africa.

Group	Species	No. Sampled (No. positive, %)					
		June 2016	January 2017	July 2017	April 2018	August 2018	Total
Fish	*Alestes* spp.	21	0	0	0	0	21
	*Barbus* spp.	4	0	0	0	0	4
	*Barbus baudoni*	1	0	0	0	0	1
	*Brycinus* spp.	3	0	0	0	0	3
	*Chrysichthys* spp.	5	0	0	0	0	5
	*Coptodon* spp.	51	0	0	0	0	51
	*Coptodon zilli*	10	0	0	0	0	10
	*Hemichromis* spp.	30	0	0	0	0	30
	*Hydrocynus* spp.	5	0	0	0	0	5
	*Labeo* spp.	9	0	0	0	0	9
	*Lates niloticus*	7	0	0	0	0	7
	*Malapterurus* spp.	1	0	0	0	0	1
	*Micralestes* spp.	5	0	0	0	0	5
	*Oreochromis* spp.	11	0	0	0	0	11
	*Oreochromis aureus*	1	0	0	0	0	1
	*Pangasius* spp.	6	0	0	0	0	6
	*Parachanna* spp.	1	0	0	0	0	1
	*Pareutropius* spp.	5	0	0	0	0	5
	*Petrocephalus* spp.	7	0	0	0	0	7
	*Pollimyrus* spp.	1	0	0	0	0	1
	*Synodontis* sp.	8	42	0	0	0	50
	Fish Total	192	42	0	0	0	234
Amphibian	*Hoplobatrachus occipitalis	17	36 (1, 2.8%)	32	28 (2, 7.1%)	12	125 (3, 2.4%)
	*Phrynobatrachus francisci	8 (1, 12.5%)	28	0	0	0	36 (1, 2.8%)
	*Ptychadena* spp.	44	47	5	0	0	96
	*Pyxicephalus edulis*	0	0	3	0	0	3
	*Rana galamensis*	2	1	2	0	0	5
	*Xenopus fischbergi*	2	1	8	0	0	11
Reptile	Lizard (*Varanus niloticus*)	0	2	0	0	0	2
	Turtle (*Pelomedusa* sp.)	0	0	2	0	0	2
	Amphibian/reptile Total	73	115	52	28	12	280

^*^Species positive for *D*. *medinensis* L3s.

We recovered a total of 863 larvae from 276 amphibians, 90 (10%) of which met morphological criteria warranting molecular identification. Of those, seven (1%) were confirmed as *D*. *medinensis* with sequences 100% similar to the previously accessioned cytochrome *c* oxidase subunit I, *D*. *medinensis* HQ216219.1 (Genbank). The first trip was conducted June-July 2016 in Marabe (Moyen Chari region, Kyabe district), and a single individual larvae was found in an African puddle frog (*Phrynobatrachus francisci*) as previously reported^6^. On two separate trips (Jan. 2017 and April 2018), we recovered six *D*. *medinensis* L3’s; two of the six were found in a single *H*. *occipitalis* bullfrog during Jan. 2017 from the village Sidi-1in the Bousso area (Fig. 1) while the remaining four were detected in April 2018 from Tarangara village in the Sarh area (three larvae from a *H*. *occipitalis* and one larva from another *H*. *occipitalis*) (Fig. 1). Additional nematodes were obtained from the amphibian samples, although we did not enumerate or identify all specimens. Of those identified, we found *Raillietnema* spp., *Cosmocercoides* spp., and Spirurida species.

**Figure 4:**
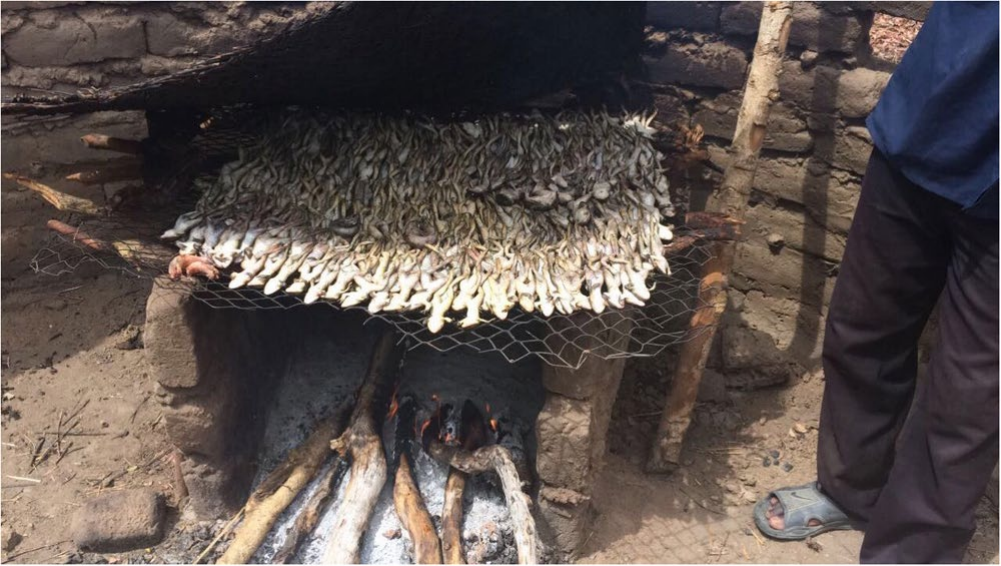
Image of frogs being cooked on grill from Doba, Moissala district, Chad, Africa taken April 2017 (photo courtesy of H. Zirimwabagabo).

When collecting samples in villages (Fig. 1), numerous villagers answered the questionnaire about frog hunting and consumption. Villagers stated that frogs were captured and consumed; however, some villagers did not eat frogs for various reasons. Two species of frogs (*H*. *occipitalis* and *P*. *edulis*) were the targets of frog catchers. When interviewed, frog catchers were able to describe both aforementioned species prior to seeing photos, then accurately picked photos of each species when shown. No one who admitted to eating frogs said that they consumed frogs raw, all stated that they cook frogs very well (Fig. 4), similar to fish, and they do not feed frogs to dogs for fear that the dogs would become sick.

## Discussion

Annual increases of GW infections since 2012 among domestic dogs and more recently cats, and the absence of classical water-borne outbreaks of GWD among humans led to the hypothesis that transmission was not occurring via the classical route of ingesting water containing copepods infected with L3s. Instead, it was posited that a paratenic host may be involved and that domestic animals were being infected via consumption of undercooked flesh or viscera of aquatic animals^3^. Our previous experimental studies showed that various amphibian species are susceptible to infections; however, in order to be important in the natural transmission cycle of *D*. *medinensis*, natural infections must occur. Thus, the goal of this study was to investigate what aquatic animals may be infected with *D*. *medinensis* L3s (as a result of ingestion/consumption of copepods containing L3s). Our intensive surveys in areas of concern in Chad identified several infected frog species including two species that are known to be eaten by people. No *D*. *medinensis* larvae were found in any of the sampled fish, *Varanus*, or turtles, although only a limited number of the latter two groups were sampled.

Many Chadian villages rely heavily on fish protein during off-seasons of agricultural production (July-November). Villages in close proximity to the Chari river that identify as fishing villages are at highest risk for *D*. *medinensis* transmission based on biological, epidemiological, and environmental investigations by the Centers for Disease Control and Prevention and The Carter Center Guinea Worm Eradication Program^2^. The absence of detection of *D*. *medinensis* L3s from any of the fish samples obtained from villages actively fishing the same species from local ponds and river tributaries suggests a limited role of fish as paratenic hosts for *D*. *medinensis*. Our detection method (using Baermann funnels and sedimentation) has been widely used in detection of larvae specimens from muscular tissues of hosts^11,12^. However, this method likely lacks sensitivity as it requires larvae to migrate out of tissues. When considering the role of fish, data from experimental studies indicate fish have been challenging to infect with *D*. *insignis* (commonly found in North American raccoons (*Procyon lotor*)), and *D*. *medinensis* infection in fish has not occurred in Chad^4,13^.

We did not recover any larvae from fish; however the importance of fish to the transmission of *D*. *medinensis* in Chad remains uncertain. Although it appears unlikely that fish act as a paratenic host in the wild, a recent study using fish species found in Chad showed that fish may serve as short term transport hosts^5^. In this situation, a transport host would be a smaller fish that feeds on copepods infected with *D*. *medinensis* L3s and is subsequently consumed by a definitive host prior to complete digestion or passage of parasites thereby “transporting” potential infection to definitive hosts^5^. Larger fish may also ingest copepods so allowing dogs or cats to ingest any fish intestines could pose a risk^5^. The methodology for larval recovery is different than that of paratenic hosts, and L3s would need to be recovered from the gastrointestinal tract. The role of fish as transport hosts in natural settings has yet to be determined and warrants investigation, especially considering increasing incidence of dog and cat infections in Chad along the Chari river corridor where there is significant fishing pressure^2^.

We recovered *D*. *medinensis* L3s from several amphibians, but the prevalence and burden of infection per individual frog was low (1–2 larvae/infected individual); however, our sampling may have missed infections due to low sensitivity of our recovery method or because infections in amphibians may be temporally and/or spatially clustered. For example, had we sampled tadpoles or frogs soon after they emerged from a water body known to have been contaminated with *D*. *medinensis*, the prevalence or worm burdens may have been higher. In North America, *D*. *insignis* has been used as a model parasite to evaluate potential transmission routes for *D*. *medinensis*. Additionally, field-based studies on *D*. *insignis* transmission have been conducted and we recently detected *D*. *insignis* L3s in several southern leopard frogs (*Lithobates sphenocephala*) at a site where prevalence of *D*. *insignis* is very high in raccoons (*Procyon lotor*) and one adult frog contained 45 larvae in its musculature (Cleveland, C.A. unpublished data). This finding of a single, heavily infected paratenic host could support the hypothesis that paratenic hosts, if ingested, could lead to infection with numerous adult nematodes in definitive hosts.

In Chad, there have been dog infections in which >50 worms emerge, but the average infection per dog is 2 worms with a range of 1–79 (The Carter Center)^14^. For those dogs with high worm burdens, a large number of L3s must be ingested, either from a fish acting as a transport host, a number of frogs acting as paratenic hosts, ingestion of water containing infected copepods, or a combination of these possible routes. Certain individual dogs may also exhibit behaviors that put them at higher risk for infection, such as preferentially predating and consuming amphibians or living in households that feed large numbers of raw fish or fish entrails to their animals. Although there are now several hypothesized alternative transmission routes, classical transmission by ingestion of water with infected copepods remains a possibility. A recent experimental study shows that dogs are capable of ingesting copepods during a normal drinking event; however, low numbers were ingested per drinking event and in cases where larger number of copepods were ingested, the density of copepods was typically higher than what is normally seen in Chadian water-bodies^15^. Instances of cat infections in Chad further complicate identifying the type of transmission occurring, and studying the diets and movement of cats that have been infected or live in villages with dog infections could be informative.

Integration of the potential transmission routes may be advanced using mathematical modeling. For example, a combination of transmission routes in avian influenza^16^ and Ebola^17^ help to explain phenomena including pathogen invasion and persistence. Uncertainty in model structure, including the existence of certain transmission routes, as well as parametric uncertainty, especially the rates and probabilities associated with transmission routes, can be explored using mathematical models in conjunction with sensitivity analyses^18^. These approaches allow an assessment of which features of transmission are most likely to interrupt the overall transmission cycle^19^. In the case of *D*. *medinensis* in Chad, transmission via transport and paratenic hosts may be combined with waterborne transmission in a model to both explore the likely impact of reducing or blocking one of these routes on overall transmission, and to estimate key parameters by fitting such models to data.

Additional data on the role of paratenic and transport hosts as food sources could also be used to strengthen model development as well as in the design and implementation of interventions in Chad. For example, *H*. *occipitalis* (Fig. 3), which was infected with *D*. *medinensis* L3s, was confirmed as a human food source through questionnaires conducted in numerous villages among various local ethnicities. Additionally, *P. edulis* (Fig. 3), was also identified as a human food source, and although we did not detect *D*. *medinensis* in this species, only three individuals of this species have been sampled to date so additional testing is needed. It is important to note that people consume frogs commonly and consistently report cooking them (Fig. 4), which would kill any parasites present. They also alleged they did not purposely feed frogs to dogs, but bringing the frogs into the village could provide a scavenging opportunity for dogs and Carter Center researchers have observed children feeding tadpoles to dogs (unpublished data). However, ingestion of both paratenic and transport hosts could occur independently of human activities during foraging and scavenging events by animals.

While we have provided evidence supporting the possibility of an alternative method of transmission for Guinea worm, our results should be considered a conservative estimate of the number of amphibians infected with Guinea worm L3s. Two of the surveys occurred outside of the peak transmission season (January-June), and may not be representative of the prevalence in amphibians or possible host diversity. An additional important consideration is that detection of *D*. *medinensis* L3s via our methods is likely insensitive, and it is possible that larvae were missed during the process of necropsy and microscopy. Also, the application of Abate® (Temephos) to control copepod populations post potential contamination of water-bodies with Guinea worm could affect the number of L3s recovered from an amphibian paratenic host or from fish that may be transport hosts.

The role of amphibians is not yet fully defined, but there are additional challenges based on life-stages of amphibians. For example, tadpoles are very likely the key life-stage wherein ingestion of infected copepods occur and if tadpoles consume large numbers of infected copepods, they could be acting as a sink for infection if tadpole/amphibian ingestion by dogs or people is ultimately shown to be low. Also, if the tadpoles consuming the copepods are from a fully aquatic amphibian species, such as *Xenopus*, then they are less likely to be scavenged or hunted by a cat or dog, despite having potential infective L3s in their tissue. Conversely, the possibility for amphibians to act as long-term paratenic hosts must be considered. Data from an experimental study showed that motile larvae of *D*. *insignis* and *D*. *medinensis* were recovered from frog tissues (*Xenopus* spp.) for up to 8 months and 2 months, respectively (Cleveland and Yabsley, unpublished data).

### Conclusions and management implications

Stopping transmission of Guinea worm and achieving eradication of *D*. *medinensis* is the goal of the global Guinea Worm Eradication Program. Alternative transmission routes for *D*. *medinensis* to humans and animals are a concern for eradication. While we have identified *D*. *medinensis* infection in frogs, we have not received reports nor collected data on dogs or cats consuming frogs. Consumption of frogs by peri-domestic animals may occur, and further studies are needed to identify the location and frequency of these behaviors. Furthermore, while our data do not provide evidence that fish are paratenic hosts, there has been no research yet conducted on the presence of *D*. *medinensis* larvae in the intestines of fish in Chad; however, large numbers of copepods have been observed in the digestive tracts of small fish (Cleveland, unpublished data). A recent case-control study conducted by The Centers for Disease Control and Prevention personnel in Chad investigating risk-factors contributing to infection with *D*. *medinensis* reported a statistically significant association between provisioning of fish entrails to dogs and infections among dogs (S. Roy, The Centers for Disease Control and Prevention, personal communication).

Understanding how transmission is occurring among dogs and cats would allow for implementation of timely and more effective preventative measures to be devised. In order to ensure compliance among village residents, the intervention(s) must be simple and straightforward. The owners of peri-domestic animals in Chad must understand the importance of limiting the ability of dogs and cats to scavenge or consume frog flesh and fish entrails. It should also be noted that occurrence of *D*. *medinensis* transmission in Chad primarily occurs along the Chari river and tributaries, but, there are few human cases and animal infections from communities closer to the Logone river (Fig. 1), a major tributary of the Chari river (H. Zirimwabagabo, The Carter Center-Chad, personal communication). Furthermore, dog, cat and baboon (*Papio anubis*) infections have been documented in Ethiopia and dog infections in Mali, suggesting that potential animal behaviors and transmission route(s) may differ^20^. Therefore, careful field investigations in each region will be essential during, what is hoped to be, the tail end of the Guinea worm eradication program.

## Data Availability

Any data discussed herein will be made available upon written request.

## References

[1] Hopkins DR, Ruiz-Tiben E, Eberhard ML, Roy SL (2015). Progress toward global eradication of dracunculiasis, January 2014–June 2015. MMWR Morb. Mortal. Wkly. Rep..

[2] Hopkins DR, Ruiz-Tiben E, Eberhard ML, Roy SL, Weiss AJ (2017). Progress toward global eradication of dracunculiasis, January 2016–June 2017. MMWR Morb. Mortal. Wkly. Rep..

[3] Eberhard ML (2014). The peculiar epidemiology of dracunculiasis in Chad. Am. J. Trop. Med. Hyg..

[4] Eberhard ML (2016). Possible role of fish and frogs as paratenic hosts of Dracunculus medinensis, Chad. Emerg. Infect. Dis..

[5] Cleveland CA (2017). Possible role of fish as transport hosts for *Dracunculus* spp. larvae. Emerg. Infect. Dis..

[6] Eberhard ML (2016). Guinea worm (*Dracunculus medinensis*) infection in a wild-caught frog, Chad. Emerg. Infect. Dis..

[7] Cleveland CA (2018). The wild world of Guinea Worms: a review of the genus *Dracunculus* in wildlife. Int. J. Parasitol. Parasites. Wildl..

[8] Wang J (2016). Phylogenetic relationships of five asian schilbid genera including *Clupisoma* (Siluriformes: Schilbeidae). PloS one..

[9] Prosser SW, Velarde‐Aguilar MG, León‐Règagnon V, Hebert PD (2013). Advancing nematode barcoding: a primer cocktail for the cytochrome c oxidase subunit I gene from vertebrate parasitic nematodes. Mol. Ecol. Resour..

[10] Mirza MB, Basir MA (1937). A report on the guinea-worm found in V*aranus* sp., with a short note on *Dracunculus medinensis*. Proc. Natl. Acad. Sci. India Sect. B. Biol. Sci..

[11] Ash LR (1968). The occurrence of *Angiostrongylus cantonensis* in frogs of New Caledonia with observations on paratenic hosts of metastrongyles. J. Parasitol..

[12] Holliman RB, Meade B (1980). Native trichinosis in wild rodents in Henrico County, Virginia. J. Wildl. Dis..

[13] Crichton VFJ, Beverley-Burton M (1977). Observations on the seasonal prevalence, pathology, and transmission of *Dracunculus insignis* in the raccoon in Ontario. J. Wildl. Dis..

[14] WHO Collaborating Center for Research Training and Eradication of Dracunculiasis, *Guinea Worm Wrap-Up #24*1, June 17, 2016, Centers for Disease Control and Prevention (CGH): Atlanta (2016).

[15] Garrett, K. B., Box, E. K., Cleveland, C. A., Majewska, A. A. & Yabsley, M. J. Dogs and the classic route of transmission: an evaluation of copepod ingestion. Manuscript submitted for publication (2018). 10.1038/s41598-020-58191-4 PMC6989452 31996759

[16] Rohani P, Breban R, Stallknecht DE, Drake JM (2009). Environmental transmission of low pathogenicity avian influenza viruses and its implications for pathogen invasion. Proc. Natl. Acad. Sci..

[17] Vinson JE, Drake JM, Rohani P, Park AW (2016). The potential for sexual transmission to compromise control of Ebola virus outbreaks. Biol. Let..

[18] Simpson JE (2012). Vector host-feeding preferences drive transmission of multi-host pathogens: West Nile virus as a model system. Proc. R. Soc. Lond. [Biol.].

[19] Fraser C, Riley S, Anderson RM, Ferguson NM (2004). Factors that make an infectious disease outbreak controllable. Proc. Natl. Acad. Sci..

[20] Molyneux D, Sankara DP (2017). Guinea worm eradication: Progress and challenges-should we beware of the dog?. PLoS Negl. Trop. Dis..

